# Deciphering the role of Wnt/β-catenin and miR-214 in knee osteoarthritis: molecular and clinical insights

**DOI:** 10.3389/fphar.2025.1507693

**Published:** 2025-02-25

**Authors:** Waad A. Samman, Esraa M. Mosalam, Dalia S. Saif, Mahmoud S. Abdallah, Abdel-Aziz A. Zidan, Amany Said Sallam, Shimaa Abdelsattar, Fatma Omar Khalil, Amany E. Elashkar, Somia Mokabel Mohamed, Mohamed Al-Ghannam, Hend E. Abo Mansour

**Affiliations:** ^1^ Department of Pharmacology and Toxicology, College of Pharmacy, Taibah University, Medina, Saudi Arabia; ^2^ Department of Biochemistry, Faculty of Pharmacy, Menoufia University, Shebin EL-Kom, Menoufia, Egypt; ^3^ Department of Pharm D, Faculty of Pharmacy, Jadara University, Irbid, Jordan; ^4^ Department of Rheumatology, Physical Medicine, and Rehabilitation Department Faculty of Medicine, Menoufia University, Shebin EL-Kom, Menoufia, Egypt; ^5^ Department of Clinical Pharmacy, Faculty of Pharmacy, University of Sadat City, Sadat City, Menoufia, Egypt; ^6^ Zoology Department, Faculty of Science, Damanhur University, Damanhour, Egypt; ^7^ Department of Pharmacology and Toxicology, Faculty of Pharmacy, Menoufia University, Shebin EL-Kom, Menoufia, Egypt; ^8^ Department of Clinical Biochemistry and Molecular Diagnostics, National Liver Institute, Menoufia University, Shebin EL-Kom, Menoufia, Egypt; ^9^ Clinical Microbiology and Immunology Department, National Liver Institute, Menoufia University, Shebin EL-Kom, Menoufia, Egypt; ^10^ Clinical Pathology Department, National Liver Institute, Menoufia University, Shebin EL-Kom, Menoufia, Egypt; ^11^ Department of Physiology, Faculty of Medicine for Girls, Al-Azhar University, Cairo, Egypt; ^12^ Department of Radiology, Faculty of Medicine, Menoufia University, Shebin EL-Kom, Menoufia, Egypt; ^13^ Biochemistry Department, Faculty of Pharmacy, Menoufia National University, Birket El-Sab, Egypt

**Keywords:** knee osteoarthritis, miR-214, Wnt/β-catenin, PGSK3β, WOMAC score

## Abstract

**Introduction:**

Understanding the molecular mechanism underlying the pathogenesis of knee osteoarthritis (KOA) may be beneficial in fetching new therapeutics. Our study aims to investigate the implication of Wnt/ β-catenin pathway in development of KOA by detection of the downstream target genes and their crosstalk with miR-214 in patients with KOA and to correlate that with the clinical findings.

**Methods:**

Sixty participants were involved in the study. The levels of miR-214, β-catenin, Wnt4, matrix metalloproteinase 3 (MMP3), Bax, caspase 3, and phosphorylated glycogen synthase kinase-3 beta (pGSK3β) were determined. All participants were assessed clinically and radiologically regarding knee joint pain, stiffness, range of motion, and knee medial cartilage thickness. Besides, a correlation between Western Ontario and McMaster Universities (WOMAC) score, clinical, and radiological data, and the measured parameters was conducted.

**Results and discussion:**

Patients with KOA showed downregulated miR-214 with upregulated β-catenin, Wnt4, MMP3, Bax, caspase 3, and pGSK3β compared to healthy individuals. Statistically significant positive correlation between WOMAC score, knee joint pain regarding Visual Analogue Scale (VAS) with β-catenin, pGSK3β, Wnt4, MMP3, Bax, and caspase 3, and significant negative relationship between them and knee joint medial cartilage thickness; while there was a statistically significant negative correlation between WOMAC, and clinical findings of osteoarthritis and miR-214 and significant positive relationship between it and knee joint medial cartilage thickness. This study provides valuable insights into involvement of the Wnt/β-catenin and miR-214 in KOA pathogenesis. By targeting these molecular components, future therapeutics may modulate their activity and mitigate chondrocyte apoptosis and matrix degradation, potentially halting KOA progression.

## Graphical Abstract

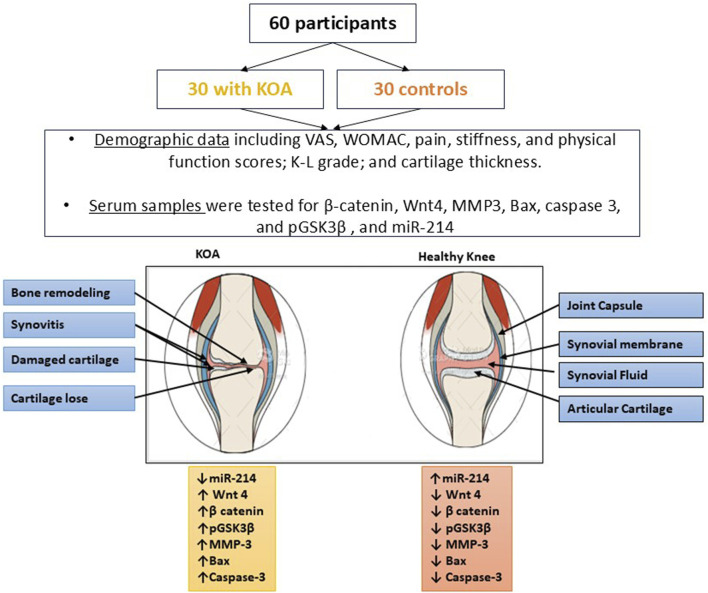

## 1 Introduction

Osteoarthritis (OA) is the most widespread form of long-lasting musculoskeletal diseases. Hip and knee OA are affecting about 300 million worldwide and they represent the 11th cause of global disability as measured by years lived with disabilities (YLDs). knee osteoarthritis (KOA) is characterized by the progressive breakdown of cartilage, which is mediated by inflammatory cytokines and other molecules (Liang et al., 2019). The stress on the knee increases as the KOA progresses because it gradually impairs the function of the knee, hurts and weakens the muscles. Also, it causes joint discomfort that develops gradually, gets worse with movement, spreads after extended periods of sitting or sleeping, and gets worse over time with stiffness, edema, restricted range of motion, and crepitus in the affected joint (Fang et al., 2021). Despite the progressive degenerative nature of the disease with aging, factors such as obesity, previous knee injuries, and regular crouching and kneeling increase the risk of early onset of knee osteoarthritis (KOA) (Blagojevic et al., 2010). Numerous studies have validated the use of ultrasonography for knee osteophytes, medial meniscal protrusion, and morphological alterations in the medial femoral condyle cartilage assessment and grading of KOA (Serban et al., 2016). When conservative treatment fails, surgical alternatives are considered as a next step in the management of KOA.

Depending on the etiology, KOA is classified as either primary or secondary. Primary KOA is caused by unidentified articular cartilage degeneration. This is frequently referred to as age-related degradation and wear and tear. Secondary KOA is caused by articular cartilage degeneration due to a known cause, such as post-traumatic reasons (Manlapaz et al., 2019). Chondrocytes and extracellular matrix (ECM) components make up articular cartilage, an avascular tissue with a restricted potential for regeneration. The sole cell type in articular cartilage is the chondrocytes which control the anabolic to catabolic ratio to preserve the healthy function of the ECM (Moo et al., 2014). Chondrocyte death throughout the OA process is linked to degradation and calcification of ECM; suggesting a role for cell death in the OA pathogenesis. It might be able to boost chondrocyte proliferation to postpone OA development and progression (Gao et al., 2014). Understanding the pathophysiology of the developmental process of OA is depending on exploring the molecular mechanisms underlying the symptoms, which include articular cartilage deterioration, formation of bone spurs, subchondral sclerosis, and synovial hyperplasia (Chen et al., 2017).

Preceding studies have linked Wnt/β-catenin signaling trajectory in the development of OA (Appleton et al., 2010; Yuasa et al., 2008). Wnt is a glycoprotein that secreted outside of the cells with ninteen Wnt genes and many Wnt receptors that govern both canonical and non-canonical-catenin-dependent signaling pathways. Various activities involving cell division, polarization, proliferation, and fate of the cells throughout embryogenesis and later stages of development, are linked to both downstream pathways (Liu S. et al., 2017); as well as the development and progression of various diseases, with growing evidence of their pathogenic involvement in OA. The attachment of Wnt proteins to their receptors inhibits the phosphorylation of β-catenin and glycogen synthase kinase 3 beta (GSK3β). Non-phosphorylated catenin accumulates in the cytoplasm and travels to the nucleus to regulate the expression of target genes, accelerate the cell cycle, and encourage proliferation of cells (Fensterle, 2006).

MicroRNAs (miRNAs) are a subclass of non-protein-coding RNA molecules, with an approximate length of 22 nucleotides. They can mediate the post-transcriptional expression of various genes for the controlling of several physiological and pathological events. However, the degree of sequence similarity between their regulatory activities and the 3′-UTR of target mRNAs varies. It has been shown that miRNA expression controls bone and cartilage growth and preserves homeostasis in the latter (Borgonio et al., 2014; Duan et al., 2020). Previous studies have examined the distinct functions of several miRNAs in KOA, concluding that the expression of miR-214 controls crucial signaling pathways throughout the development of OA, such as the Wnt/β-catenin signaling (Cao et al., 2017). In this work, we aim to explore the possible role of miR-214 in KOA pathogenesis and diagnosis and also to establish a correlation between miR-214 expression and Wnt/β-catenin, clinical and radiological findings in patients with KOA in an attempt to provide molecular basis for developing novel miRNA-based targeted treatment modalities.

## 2 Patients and methods

### 2.1 Patients

A case-control study was conducted at Menoufia University Hospital’s Department of, Rheumatology, Rehabilitation, and Physical Medicine with 30 KOA cases with average age of 57.57 years and 30 controls with matched sex. Patients were diagnosed using the American College of Rheumatology criteria, which included primary KOA with Kellgren-Lawrence grade (K-L grade) ≥ 1 (Mahmoudian et al., 2021).

One consultant, blind to the clinical data, performed knee ultrasonography within the same day as the clinical and radiographic, and laboratory tests. The physician used a (LOGIQ- E10) fitted with a high frequency broadband linear array transducer at 10–12 MHz. The medial cartilage thickness was assessed: When the patients were supine, their knees were in maximum flexion and the probe was put in an axial plane on the suprapatellar region then femoral cartilage was measured. The cartilage degeneration was categorized semi-quantitatively as follows: 0: normal, grade-1 (mild OA): loss of normal sharpness of cartilage interfaces and/or increased echogenicity of the cartilage, grade-2A (moderate OA): strong local thinning (less than 50%) of the cartilage, grade-2B (moderate OA): local thinning of the cartilage more than 50% but less than 100%, and grade-3 (marked OA): 100% local loss of the cartilage (Schiphof et al., 2008; Østergaard and Wiell, 2004; Boonstra et al., 2008).

The following criteria were excluded: gouty arthritis, post-traumatic arthralgia, autoimmune or connective tissue diseases, metabolic disorders, inflammatory arthritis, endocrine abnormalities, and developmental dysplasia. Healthy controls with similar age and gender were selected among hospital employees and individuals receiving routine medical examinations. Based on the medical record, no control ever experienced any clinical symptoms or signs (pain, swelling, tenderness, or restriction of movement) of OA, other arthritis, or joint illnesses at any site. No controls were related to the patients or the OA history in their family. Furthermore, they did not have any risk factors for KOA that could confound results, such as body mass index (BMI) equal to or more than 30 according to the WHO criteria, menopausal status, estrogen therapy, and type II diabetes mellitus.

Every participant’s demographic, lifestyle, and clinical information—including age, gender, BMI, and K-L grading were documented. In the case of OA, the pain was measured using the Visual Analogue Scale (VAS) with ten scale bars (0–10) on one side (Boonstra et al., 2008; Alexander, 2007). As a measure of the severity of a condition, the Western Ontario and McMaster Universities (WOMAC) Osteoarthritis Index scale was used and each patient who was enrolled completed the validated Arabic version of the WOMAC score questionnaire (Faik et al., 2008), which includes 24 items measuring 3 subscales including: the primary factors used to assess the alterations in the structure and function of the knee joint regarding: pain (5 items) with pain ranging from 0 to 20 points, stiffness (2 items) that ranged from 0 to 8 points, and physical function (17 items) ranged from 0 to 68 points. Scores can be calculated for each subscale and a total WOMAC score is calculated by adding all 3 subscale scores that ranged from 0–96 points where 0 represents the best health status and 96 the worst possible status.

All participants provided written informed consent before participation. The Ethical Committee of the Faculty of Medicine Hospital at Menoufia University, under IRB number 12/2022RAD18, accepted the research protocol. The ethical approval complied with the Declaration of Helsinki’s requirements.

### 2.2 Sample size calculation

Based on a review of past literature by Cao et al. (2021a) who reported that miR-214-3p expression was downregulated in inflamed chondrocytes and KOA cartilage. A minimum of sixty participants was determined to be the sample size. G Power software was used to divide them equally into two main groups, each with thirty participants at 80% power and 95% confidence intervals.

### 2.3 Measurements

All patients' blood was collected and put in vacuum-sealed containers for centrifugation at 4500 g for 15 min. Serum samples were placed into Eppendorf tubes and stored at −80°C for analyses. Specific commercial ELISA kits (MyBioSource, Inc.) were used to determine β-catenin, Wnt4, matrix metalloproteinase 3 (MMP3), Bax, and caspase 3 according to the manufacturer’s instructions with catalogue number MBS8291331, MBS8802754, MBS045298, MBS2701194, MBS2513810; respectively. The level of pGSK3β (Ser9) was also measured using a commercially available ELISA kit (Cat No. PEL-GSK3b-S9, RayBiotech, Inc., United States) according to the supplier’s instructions.

### 2.4 miRNA assay

miRNeasy serum/plasma kit (QIAGEN, Germany, Cat. No. 217184) was used to extract total RNAs, including miRNAs, from serum samples in accordance with the manufacturer’s instructions. The quantity of RNA present was quantified using a Multiskan Sky High Microplate Spectrophotometer from Thermo Fisher Scientific (United States). By using qRT-PCR, the expression of miR-214 was detected. Thermo Fisher Scientific’s TaqMan^®^ microRNA reverse transcriptase kit (Cat. No. 4366596) and the TaqMan assays for hsa- miR-214 and RNU6B (Assay IDs: 002293 and 001093, respectively) were used to convert RNA to cDNA. miR-214 expression was compared to RNU6B, an endogenous control. The PCR reactions were carried out by using TaqMan^®^ Universal Master Mix II (Applied Biosystems ™, United States, Cat. No. 4440043) in 20 μL total volume, including 10 μL TaqMan^®^ Universal Master Mix II, 1 μL TaqMan^®^ assay 20X, and 9 μL cDNA + RNase-free water. The following were the PCR settings: 1 min at 95°C, forty cycles of 95°C for 15 s, 1 min at 60°C, and 5 min at 72°C as the last extension. Real-time PCR was carried out using the StepOnePlus™ Real-Time PCR system (Thermo Fisher Scientific, United States) and the fold change and Ct were computed.

### 2.5 Protein-protein interaction (PPI) network analysis

This was created by using Cytoscape v3.10.2 and string online database; https://string-db.org/. Wnt protein was chosen as a central protein to create the network. String protein query was used in the setting of the software with a medium confidence score of 0.4, average local clustering coefficient equal to 0.878, PPI enrichment *p*-value <1.0e^−16^, and with no more than 5 interactions in the first and the second shells.

### 2.6 Statistical analysis

The mean and standard deviation (SD) for all data is displayed. Using an independent sample t-test, differences across groups were evaluated. For categorical data, the chi-square test or Fisher’s exact test was used as appropriate. The link between the measured parameters was evaluated by calculating Pearson’s correlation. A *p* value of less than 0.05 was used to determine statistical significance. The diagnostic cutoff points were chosen from the receiver operating characteristic (ROC) curve with the maximum accuracy. The computations were completed using IBM’s SPSS 21.0 statistical program (IBM, United States).

## 3 Results

### 3.1 Demographic and clinical features

The demographic and clinical characteristics of the study participants, categorized by groups and grades, are outlined in Table 1.

**TABLE 1:** Demographic and clinical features of study groups. Sixty participants were involved; 30 with KOA and 30 matched controls.

Laboratory parameters	Group 1 (KOA patients) n = 30	Group 2 (healthy control) n = 30	Statistical values
Age (years)	57.57 ± 4.38	29 ± 4.5	*P* = 0.295
Sex			*P* = 1
Male (n)	7	7	
Female (n)	23	23	
Weight (kg)	82.067 ± 3.35	70.1 ± 4.9	*P* = 0.125
Height (cm)	167.93 ± 3.3	169.66 ± 4.4	*P* = 0.072
BMI (Kg/m^2^)	29.1 ± 1.03	24.35 ± 1.65	*P* = 0.666
K–L grade	G1, G2	G0	
Medial cartilage thickness	0.746 ± 0.123	2.49 ± 0.257	*P* < 0.001
VAS score	6.6 ± 1.19	1.1 ± 0.31	*P* < 0.001
WOMAC score	57.57 ± 4.38	1.07 ± 0.37	*P* < 0.001
Pain score	7.73 ± 0.98	1.5 ± 0.5	*P* < 0.001
Stiffness score	7.167 ± 0.53	1.33 ± 0.48	*P* < 0.001
Physical function score	33 ± 2.12	8.47 ± 1.25	*P* < 0.001

KOA: Knee osteoarthritis, WOMAC, score: Western Ontario and McMaster Universities score, VAS:Visual analogue scale, BMI: Body mass index.

### 3.2 Effect on biological markers

**FIGURE 1:**
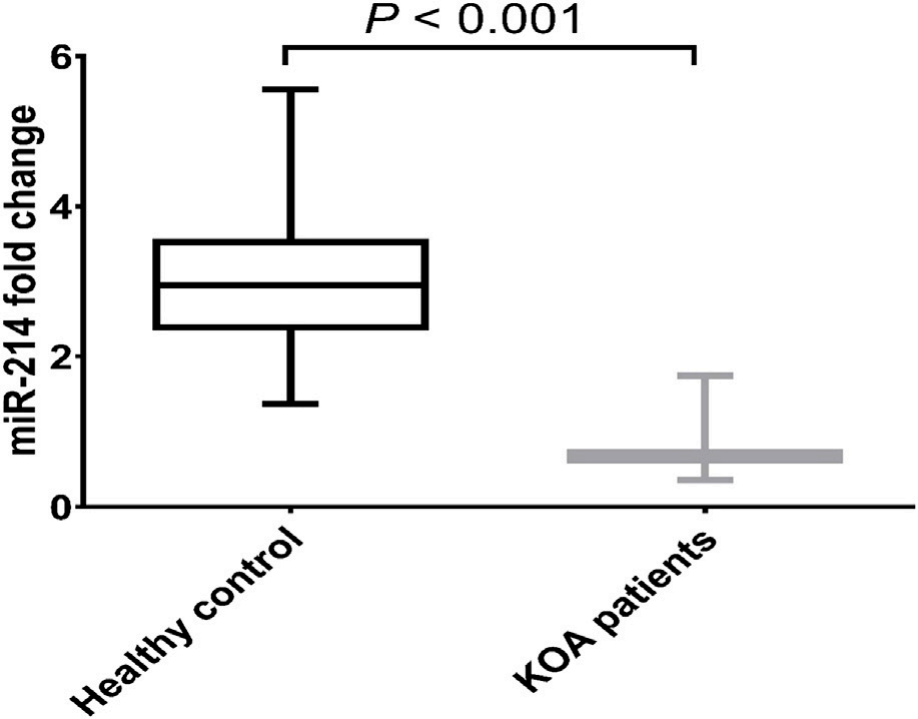
Expression pattern of miR-214 in health donors and patients with KOA. Data are presented as mean ± SD, *p* < 0.001, n = 30. miR-214 was found to be downregulated in KOA patients compared to the healthy controls.

We looked at the expression of miR-214 in serum samples either from KOA patients or healthy donors without any joint conditions to probe the potential function of miR-214 in human KOA. Comparing with healthy donors, the expressional level of miR-214 was significantly reduced in KOA patients by 75.79% (*p* < 0.001) as presented in Figure 1.

**FIGURE 2:**
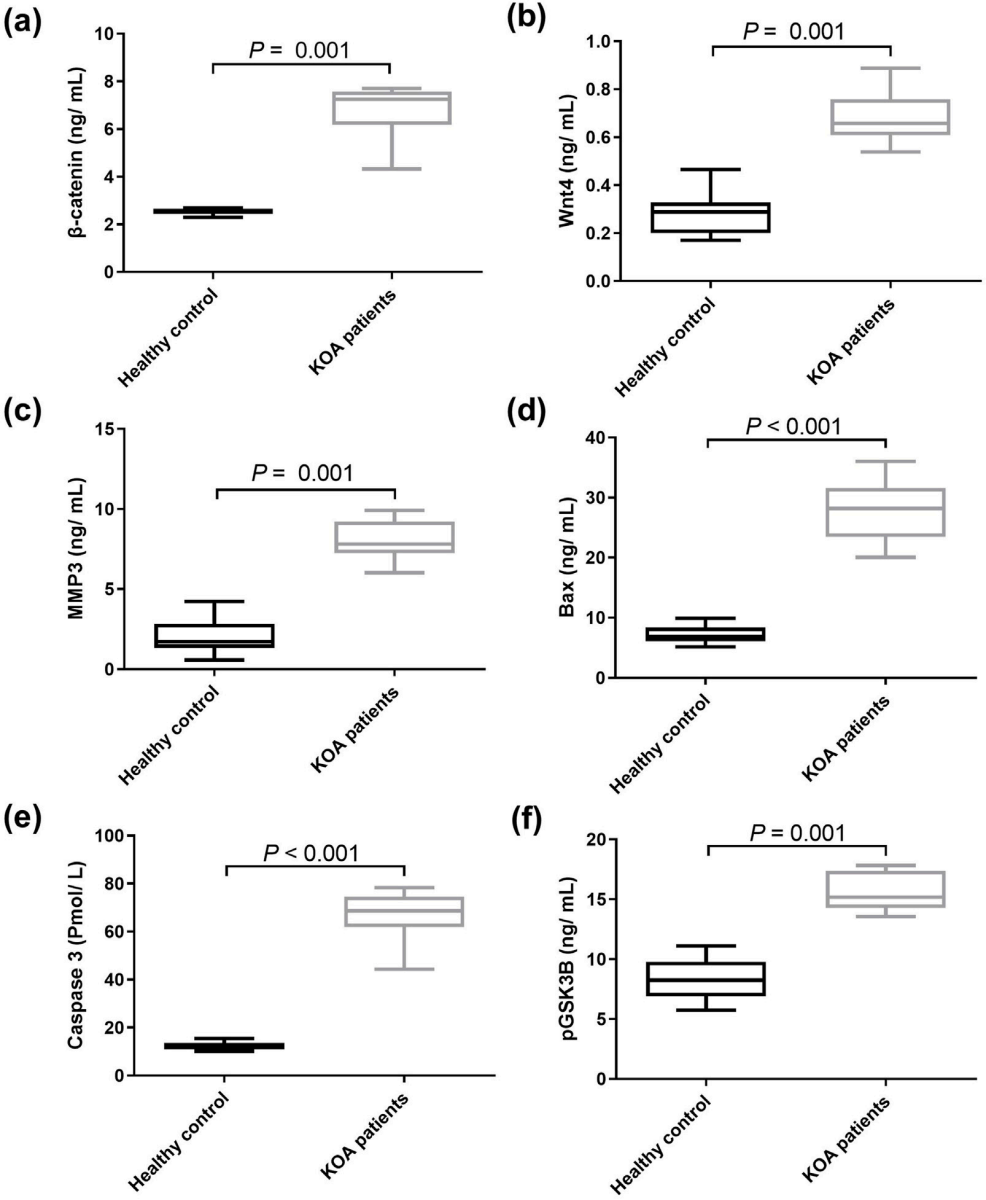
Serum levels of downstream genes of Wnt/β‑catenin signaling pathway in health donors and patients with KOA. **(A)** β‑catenin, **(B)** Wnt4, **(C)** MMP3, **(D)** Bax, **(E)** caspase 3, and **(F)** pGSK3β. Data are presented as mean ± SD, *P* < 0.01, n = 30. Wnt4: wingless-type mmtv integration site family member 4, MMP3: matrix metalloproteinase 3, pGSK3β: phosphorylated glycogen synthase kinase 3 beta. The detected genes were found to be upregulated in KOA patients compared to the healthy controls.

The protein levels of Wnt/β-catenin mediators were then examined in both healthy donors and KOA patients. Serum from KOA patients had significantly higher protein levels of β-catenin, Wnt4, MMP3, Bax, caspase 3, and pGSK3β than serum from healthy donors by 1.69, *p* = 0.0001; 1.45, *p* = 0.0001; 2.91, *p* = 0.0001; 2.88, *p* < 0.0001; 4.41 folds, *p* < 0.0001; respectively, and by 86.81%, *p* = 0.0001 for pGSK3β as shown in Figure 2.

### 3.3 Correlation study

**FIGURE 3:**
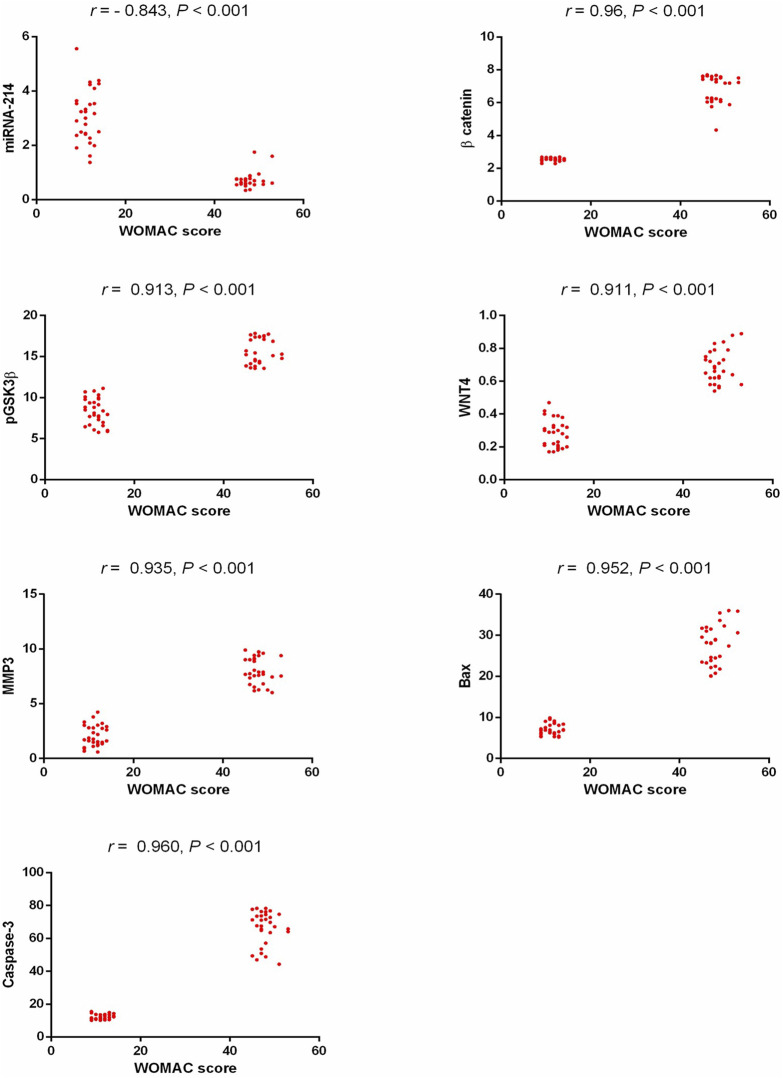
Correlation study between the detected biomarkers and WOMAC score in patients with KOA. WOMAC: Western Ontario and McMaster Universities, pGSK3β: phosphorylated glycogen synthase kinase 3 beta, Wnt4: wingless-type mmtv integration site family member 4, MMP3: matrix metalloproteinase 3, Bax: Bcl2-associated X protein.

Our results showed a statistically significant positive correlation between WOMAC scores (regarding pain, stiffness, and physical function) and β-catenin, pGSK3β, Wnt4, MMP3, Bax, and caspase 3, while there was a statistically significant negative correlation between WOMAC scores and miR-214 as shown in Figure 3. miR-214 showed a statistically significant negative correlation with β-catenin, pGSK3β, Wnt4, MMP3, Bax, and caspase 3 (r = −0.819, *p* < 0.001, r = −0.780, *p* < 0.001, r = −0.766, *p* < 0.001, r = − 0.791, *p* < 0.001, r = −0.831, *p* < 0.001, and r = −0.822, *p* < 0.001, respectively).

As shown in Table 2, there was a significant negative correlation between the serum concentration of miR-214 and the clinical finding of KOA regarding the grade of knee joint pain according to VAS, as its serum concentration was low among patients with high grades of knee joint pain, while β-catenin, pGSK3β, Wnt4, MMP3, Bax, and caspases3 concentration were positively correlated with the grade of knee joint pain regarding VAS. On another side there was a significant negative correlation between β-catenin, Wnt4, MMP3, Bax, Caspases3, and pGSK3β with the radiological data of KOA as regards to the medial cartilage thickness of the knee joint as their serum concentration was high among patients with advanced grades of OA (with lower cartilage thickness), while miR-214 serum concentration was low among patients with advanced grades of KOA and low cartilage thickness, as its serum concentration was positively correlated to medial cartilage thickness of the knee joint.

**TABLE 2:** Correlation between clinical findings regarding visual analogue scale and radiological data regarding medial cartilage thickness and lab parameters among participants of the study groups.

	VAS		Medial cartilage thickness	
	*r*	*P*	*r*	*p*
miR-214	−0.83	<0.001	0.47	<0.001
β-catenin	0.93	<0.001	−0.55	<0.001
Wnt4	0.88	<0.0013	−0.39	0.002
MMP3	0.93	<0.001	−0.47	<0.001
Bax	0.89	<0.001	−0.43	<0.001
Caspases3	0.94	<0.001	−0.49	<0.001
pGSK3β	0.92	<0.001	−0.56	<0.001

Wnt4: wingless-type mmtv integration site family member 4, MMP3: matrix metalloproteinase 3, Bax: Bcl2-associated X protein, pGSK3β: phosphorylated glycogen synthase kinase 3 beta.

### 3.4 ROC curve analysis for miR-214

**FIGURE 4:**
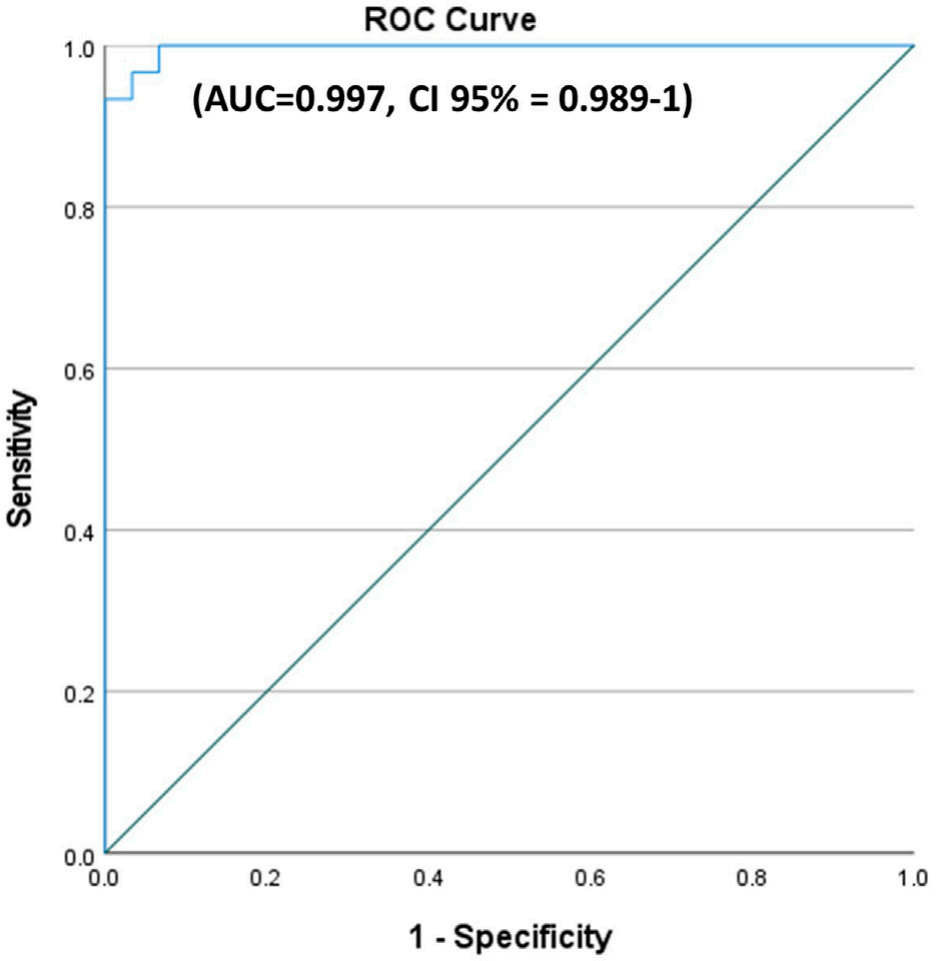
ROC curves for miR-214 in KOA patients versus healthy control. A ROC curve showed the best cutoff value of miR-214 among KOA patients to be less than 1.4832, with a sensitivity of 96.7% and a specificity of 93.3%.

A ROC curve showed the best cutoff value of miR-214 among KOA patients to be less than 1.4832, with a sensitivity of 96.7% and a specificity of 93.3% (area under the curve = 0.997; Figure 4.

### 3.5 Protein-Protein interaction network analysis

**FIGURE 5:**
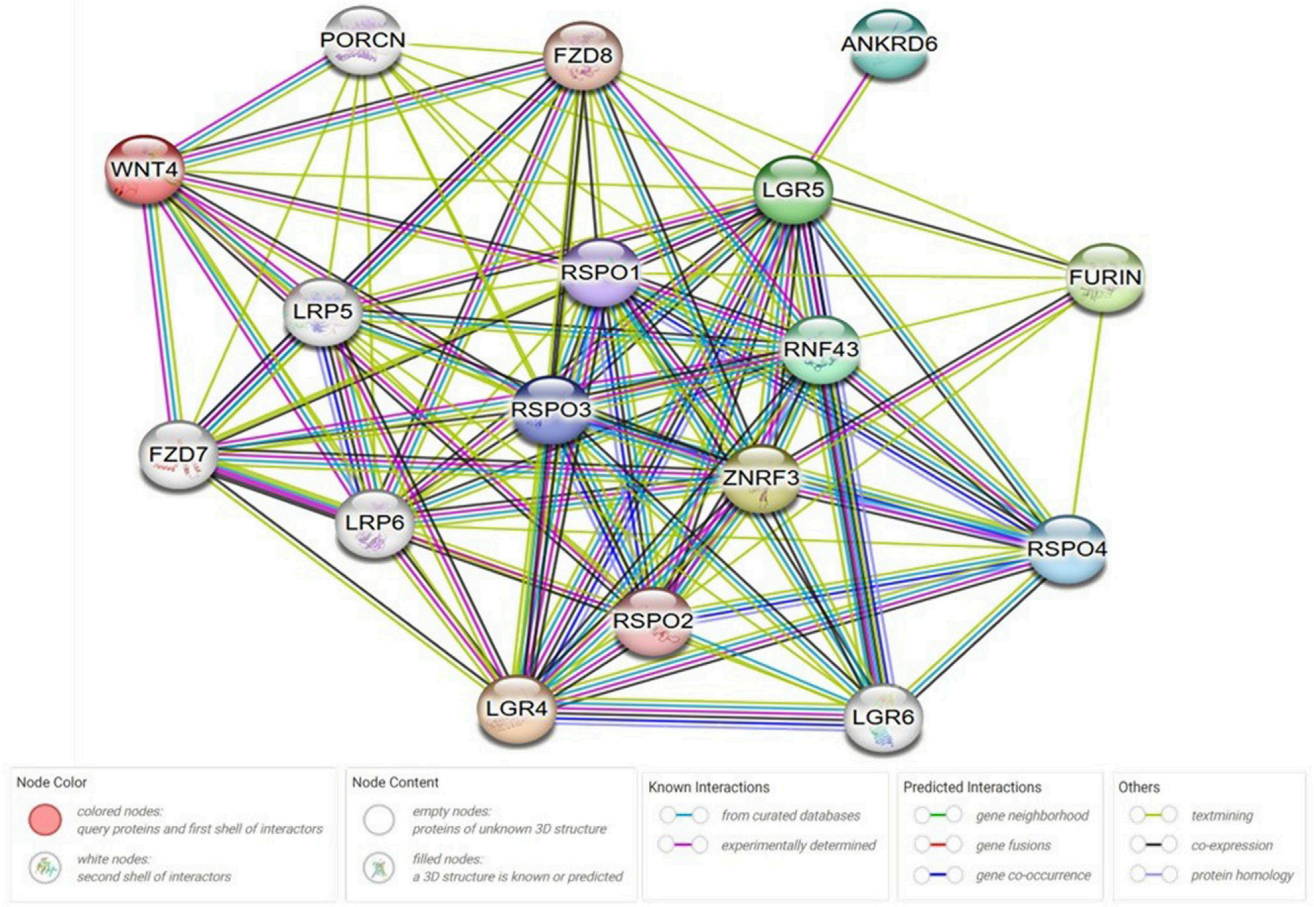
Protein-protein interaction (PPI) network analysis. The network was created by using Cytoscape v3.10.2 and string online database; https://string-db.org/. Wnt protein was chosen as a central protein to create the network. String protein query was used in the setting of the software with a medium confidence score of 0.4, average local clustering coefficient equal to 0.878, PPI enrichment *p*-value <1.0e^−16^, and with no more than 5 interactions in the first and the second shells. ANKRD6: Ankyrin Repeat Domain 6, RNF43: Ring Finger Protein 43, RSPO: R-spondin, LGR: Leucine-rich repeat-containing G-protein coupled receptor, ZNRF3: Zinc And Ring Finger 3, PORCN: porcupine O-acyltransferase, FZD: Frizzled Class Receptor.

The network created by Cytoscape revealed 17 molecular target protein that are crossly connected with Wnt/β-catenin. These protein can be further investigated in KOA to establish novel diagnostic and therapeutic avenues (Figure 5).

## 4 Discussion

KOA is the most prevalent joint condition in the world and a chief contributor to disability. KOA can have a detrimental effect on a person’s physical and mental health because it is characterized by articular cartilage loss, juxta-articular inflammation, and alterations in periarticular and subchondral bone (Stampella et al., 2017). KOA patients frequently exhibit signs of knee joint swelling, discomfort, and restriction of mobility. Hence, their quality of life is negatively impacted, and cellular inflammatory factors contribute significantly to the development of KOA (He et al., 2021). However, there are currently no efficient strategies for cartilage healing; therefore, patients with severe KOA typically require joint replacement. Understanding the pathophysiology of KOA may lead to transformative therapeutics (Wu et al., 2022). Our study is the first to establish the relationship between the expression of miR-214 and the Wnt/β-catenin signaling-related proteins and also with the clinical findings in patients with KOA; the issue which could help in better knowledge of the pathophysiology of the disease and developing novel miRNA-based treatment avenues.

In the present study and in KOA patients, we noticed a decline in miR-214 and an increase of β-catenin. This finding suggests that low miR-214 and high β-catenin levels may be linked to the pathophysiology of cartilage degeneration. Additionally, we observed a notable inverse correlation between the concentration of miR-214 in the bloodstream and the WOMAC scores, which assess pain, stiffness, and physical function in patients with KOA. Furthermore, we found that the serum concentration of miR-214 was lower in patients with higher grades of knee joint pain, as indicated by the VAS, and conversely, higher in patients with lower levels of knee joint pain. Moreover, there was a positive relationship between miR-214 concentration and the thickness of the medial knee cartilage, as determined by musculoskeletal ultrasound. Specifically, patients in advanced stages of OA with low cartilage thickness exhibited reduced levels of miR-214 in their serum, while those with higher cartilage thickness displayed elevated miR-214 levels. These findings contradict those of He et al. (2021) who suggested that miR-214 may contribute to cartilage and subchondral bone degradation, and contribute to the progression of KOA.

We could explain the controversy between the results of a study conducted by He et al. (2021) and our findings; as the study performed by He et al. (2021) was performed on patients with KOA with no healthy volunteers. It also aimed to study the effect of etoricoxib on miR-214 and inflammatory responses in those patients. This former study showed that etoricoxib has a lowering effect on miR-214 and on the synthesis of prostaglandins, with a subsequent reduction in the level of serum inflammatory biomarkers, and inhibition of some enzymes involved in the deterioration of the cartilages. They also concluded that etoricoxib has a satisfactory therapeutic effect on patients with KOA, and can notably reduce the expression of serum miR-214, which is worthy of clinical application.

On the other hand, Yuan et al. (2019) demonstrated that miR-214 prevented KOA in rats and exerted chondroprotective effects by stimulating the mitogen-activated protein kinase (MAPK) signaling pathway and as its high levels was associated with high cartilage thickness. Furthermore, Cao et al. (2021a) found that reduced miR-214 stimulates nuclear factor kappa-light-chain-enhancer of activated B cells (NF-κB) pathway and aggravates osteoarthritis progression through targeting inhibitory kappa B kinase subunit beta (IKKβ) with subsequent progression of clinical signs and symptoms of OA regarding joint pain, swelling, limited range of motion. These outcomes suggested that targeting miR-214 could be a promising new treatment approach for treating KOA patients. However, the specific functions of miR-214 in osteoarthritis and the molecular mechanisms driving its effects remain to be fully understood.

Lai et al. (2023) also, shown in their research that exosomes produced from synovial fluid that target the miR-214 anti-inflammatory pathway may inhibit the release of inflammatory cytokines that are implicated in the development of osteoarthritis, with subsequent improvement of its clinical manifestations regarding pain, swelling and limited range of motion. Also, they documented that treatment with miR-214 significantly alleviated the loss of proteoglycans, surface irregularities, synovial joint inflammation, and suppressed cartilage tissue degeneration in OA mice. It can also significantly prevent the degeneration of cartilage tissue. It was established that mice with deteriorated cartilage had lower levels of miR-214 expression in their articular cartilage, and that mice with high levels of miR-214 expression in their articular cartilage had a chondroprotective effect against the development of OA (Cao et al., 2022).

It is also noteworthy to discuss the controversial protective effect of miR-214 for the cartilage but inhibitory effect on the bone. In OA, miR-214-3p has been identified as a protective agent against cartilage degeneration. Studies indicated that miR-214-3p expression is reduced in OA chondrocytes (Fioravanti et al., 2021). Overexpression of miR-214-3p counteracts interleukin-1β (IL-1β)-induced apoptosis and matrix degradation in chondrocytes (Fioravanti et al., 2021; Cao et al., 2021b). *In vivo* studies showed that administering miR-214-3p agomir *via* intra-articular injection in OA mice reduced cartilage degeneration and synovial inflammation (Cao et al., 2021b). These results indicate that miR-214-3p has a protective function in cartilage metabolism and may halts the progression of OA. Conversely, miR-214 acts as an inhibitor of bone formation. Research shows that miR-214 inhibits osteoblast activity and deposition of the minerals in the bone matrix by targeting activating transcription factor 4 (ATF4) (Wang et al., 2013).In mouse models, upregulation of miR-214 led to reduced rates of bone formation; revealing its role in defeating osteoblast activity. Additionally, osteoclast-derived exosomal miR-214-3p has been found to inhibit osteoblastic bone formation (Li et al., 2016). Former studies suggested that miR-214-3p transfers from osteoclasts to osteoblasts, resulting in decreased bone development (Li et al., 2016; Liu J. et al., 2017). These contrasting roles of miR-214 highlight its tissue-specific functions, offering insights into potential therapeutic targets for conditions like osteoarthritis and osteoporosis.

Turning to the value of miR-214 as a diagnostic tool in KOA, the optimal cutoff value for miR-214 in the ROC curve analysis was determined to be below 1.4832, showing an impressive sensitivity of 96.7%, specificity of 93.3%, and an AUC of 0.997. Although research regarding the role of miR-214 in KOA is limited, our findings are in harmony with previous studies investigated the diagnostic potential of plasma levels of miR-200c-3p, miR-100-5p, and miR-1826 in osteoarthritis. The plasma miR-200c-3p had an AUC of 0.755, with sensitivity and specificity both at 82.00%. For miR-100-5p, the AUC was 0.845, sensitivity was 82.00%, and specificity was 86.00%. Likewise, plasma level of miR-1826 showed an AUC of 0.749, with sensitivity at 82.00% and specificity at 86.00% (Lai and Cao, 2019; Ghafouri-Fard et al., 2021).

Generally, the Wnt/β-catenin trajectory controls chondrocyte differentiation and development, and aberrant expression of components of this pathway is significantly associated to KOA (Shi et al., 2021), which suggests that Wnt signaling plays a key role in the disease pathogenesis. Consequently, the Wnt signaling is thought to be a possible target to regulate KOA (Stampella et al., 2017). It has been found that miRNAs have a role in regulating Wnt/β-catenin signaling (Cao et al., 2017). The canonical Wnt pathway starts its signaling within cells by adjusting the intracellular concentration and subcellular location of β-catenin (Chen et al., 2017). Moreover, miR-214 has been shown to regulate β-catenin (Čermák et al., 2021). Furthermore β-catenin gene was confirmed to be a direct target of miR-214 (Cao et al., 2017). In humans, at least 10 frizzled (Fzd) receptors and 19 Wnt proteins have been found. Among these, Wnt4 is expressed in growing joints and contributes to the synovial joint’s interzone growth (Corr, 2008). Wnt4 is a canonical Wnt ligand, and it has been considered to be an activator of the canonical Wnt signaling pathway (Zhu et al., 2009). Wnt ligands bind to their trans-membrane receptor Fzd and co-receptors low-density lipoprotein receptor-related protein (LRP)-5/6, which inhibit GSK3β. This boosts the amount of β-catenin, which then translocates into the nucleus to interact with the transcription factor family T-cell factor/lymphoid enhancer-binding factor (TCF/LEF), which in turn promotes the expression of particular genes (Stampella et al., 2017; Liu et al., 2019; Lietman et al., 2018). Consistent with the previous studies, our study, found that the expression of Wnt4 and β-catenin was obviously higher in KOA patients than normal ones; also, their levels were higher among patients with high WOMAC scores (regarding pain, stiffness, and physical function), high grades of pain regarding VAS, and among those with low cartilage thickness regarding ultrasonography (as their serum concentration was positively correlated to WOMAC scores, clinical finding as regards to pain, and negatively correlated to radiological findings as regard to medial cartilage thickness). These results indicated that Wnt/β-catenin signaling pathway might be activated. Besides, correlation between miR-214 and Wnt4 as well as β-catenin was analyzed; the expression of Wnt4 and β-catenin in KOA patients was shown to be adversely correlated with the expression of miR-214. This is consistent with that miR-214 inhibits human mesenchymal stem cells differentiation into osteoblasts through targeting β-catenin (Li et al., 2017). Also, miR-214 negatively regulates Wnt/β-catenin signaling in breast cancer (Yi et al., 2016).

A former study documented that throughout the process of osteogenesis, miR-214 prevented the differentiation of mesenchymal stem cells into osteoblasts through targeting β-catenin (Li et al., 2017). Buchtova et al. suggested that the Wnt/β-catenin cascade could regulate the differentiation of chondrocytes. Moreover, overactivated Wnt/β-catenin pathway causes OA-similar alterations in chondrocytes, which are principally revealed by upregulation of MMP-13 and high apoptotic rate of chondrocytes (Cao et al., 2017). Consequently, the miR-214 may have a protective effect for the cartilage *via* targeting β-catenin.

GSK3 plays a vital role in the Wnt/β-catenin signaling pathway. Under normal conditions without Wnt signaling, GSK3β has the ability to phosphorylate β-catenin. Subsequently, the phosphorylated β-catenin can be realized through E3 ubiquitin ligase, resulting in β-catenin destruction by the proteasome. As a result, the β-catenin transcriptional targets are not activated (Chen et al., 2014). Nevertheless, GSK3β′s activity is decreased by phosphorylation following Wnt signal activation, which decreases its capacity to degrade β-catenin (Gu et al., 2019). In our study, we observed an increase in the level of pGSK3β in KOA patients following the inhibition of miR-214, as compared to the control group. Moreover, the serum concentration of pGSK3β showed a positive correlation with WOMAC scores and clinical findings related to pain in KOA, while exhibiting a negative correlation with radiological findings concerning the thickness of the medial cartilage.

The Wnt/β-catenin signaling pathway was also discovered to play an important role in exacerbating KOA by overproduction of MMPs, which resulted in proteoglycan matrix breakdown. It has been demonstrated that β-catenin overexpression in mature chondrocytes can exacerbate cartilage degradation, promote chondrocyte hypertrophy, and upregulate the production of MMPs (Xu et al., 2016). MMP-3, MMP-13, MMP-7, and MMP-9 genes are among the downstream target genes for transcription that are activated when β-catenin is carried to the nucleus and attaches to TCF/LEF. All of these genes are abnormally activated in KOA and play a role in the onset and progression of the disease (Zhu et al., 2009). MMP-3 is the most important MMP family member implicated in cartilage breakdown, and it was assumed to have wide substrate specificity, allowing it to be active against collagen types II, III, and IV, as well as proteoglycans and fibronectin. Furthermore, MMP-3 can activate MMP-1, MMP-2, MMP-9, and MMP-13 (Tong et al., 2017). Therefore, one of the most important ways to prevent the development of MMP-3 and prevent chondrocyte destruction is through the efficient control of the Wnt/β-catenin signaling pathway. In this study, with the decrease of miR-214, the expression of MMP-3 was increased in KOA patients, and its serum concentration was higher among patients with higher grades of pain regarding VAS, higher WOMAC scores, and among those with lower cartilage thickness.

Chondrocytes are the sole cellular type seen in articular cartilage, and their death has been considered a potential pathological etiology. Apoptosis was more prevalent in KOA cartilage than in normal cartilage (Miao et al., 2019). Bax is a pro-apoptotic protein of Bcl-2 family that plays a critical role in the execution of the apoptotic cascade (Uren et al., 2017). Caspase-3 was thought to be involved in several types of chondrocyte apoptosis by initiating and carrying out cell death (Xia et al., 2020). In the current work, miR-214 diminished while Bax increased, resulting in caspase-3 protein activation and, eventually, chondrocyte apoptosis. The serum concentrations of Bax, and caspase-3 protein were positively correlated to WOMAC scores, clinical findings of KOA as regards to pain and negatively correlated to radiological findings as regards to medial cartilage thickness. These findings in consistent with previous study that revealed the necessity of miR-214 for the homeostasis of chondrocytes and the ECM. miR-214 overexpression was associated with lower expression of apoptosis-promoting genes such as Bax and caspase-3.

To highlight the importance of our study, this work focuses on the molecular mechanism of KOA. miR-214 was identified as a downregulated molecule, clearing up its role in the disease progression and its restoration may counteract the dysregulated Wnt/β-catenin pathway, which contributes to chondrocyte apoptosis and cartilage damage. This illustrates a crosstalk between the miR-214 and Wnt/β-catenin trajectory and explaining how molecular imbalances drive cellular and articular changes in KOA. Consequently, the current study supports developing miRNA-based treatment strategies particularly for upregulation of miR-214.

## 5 Limitations of the study

The small sample size of the present study and the recruitment of OA patients from a single medical center could be the two main limitations of this study. These limitations can also be attributed to the high cost of laboratory tests used in the study. However, we recommend conducting similar research with a larger number of patients from different medical centers. Moreover, conducting further advanced molecular techniques will be an added value to the work.

## 6 Conclusion

**FIGURE 6:**
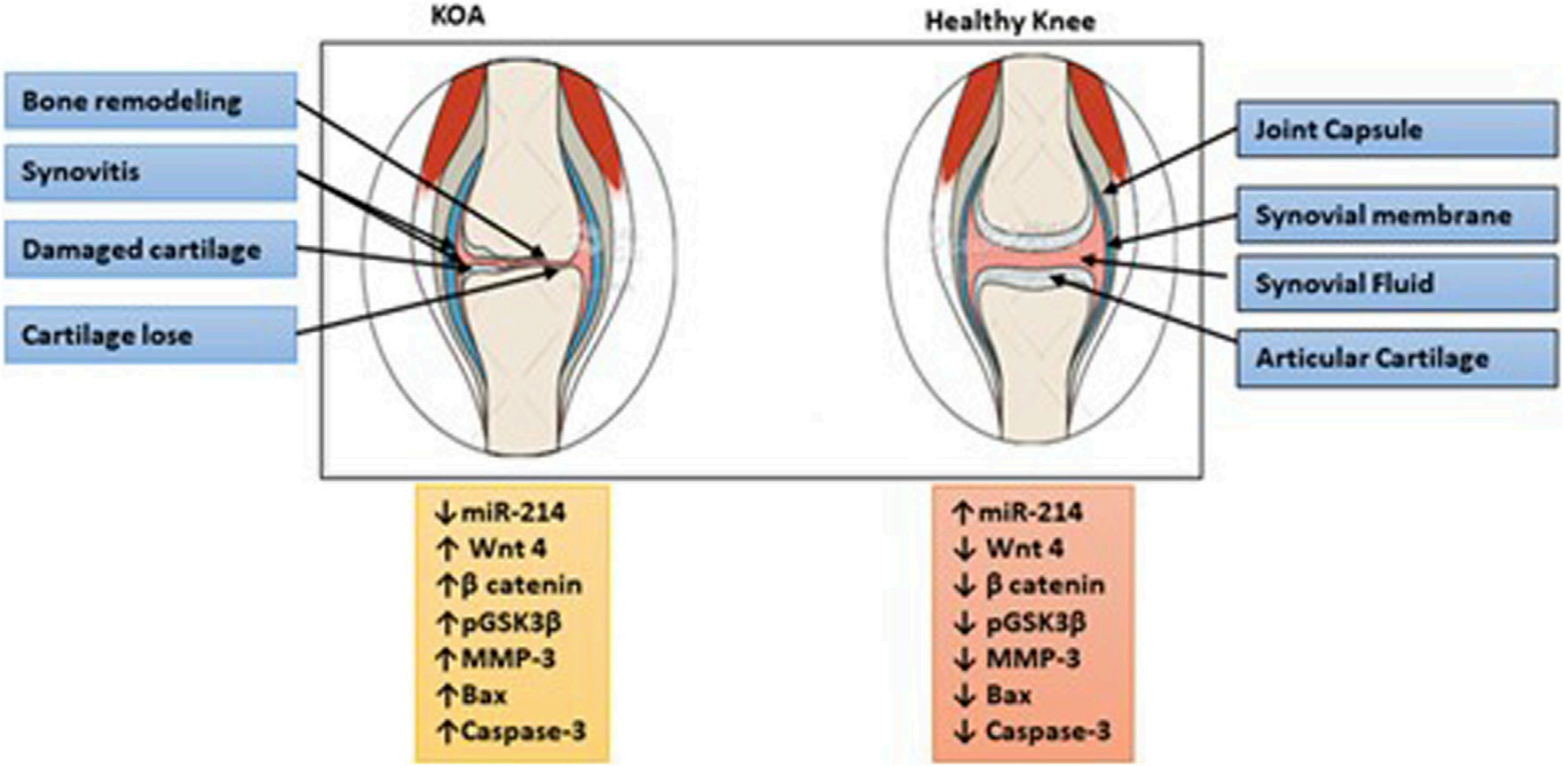
Illustration of the summary of the study. The downregulation of miR-214 is involved in the deterioration of cartilage and subchondral bone in the advancement of KOA by activating the Wnt/β-catenin signaling pathway. Individuals diagnosed with KOA experienced symptoms such as pain and stiffness in the joints, along with a reduction in the thickness of their cartilage. Conversely, elevated levels of miR-214 expression have a protective effect on cartilage and help prevent the onset of KOA.

This research contributes to understanding the complex molecular networks involved in KOA pathogenesis. Identification of miR-214 as a downregulated molecule in KOA patients suggests its potential as a promising therapeutic target for the development of novel treatment strategies. Restoring or increasing miR-214 expression may assist to overcome abnormal Wnt/-catenin pathway activation, thereby mitigating chondrocyte apoptosis and matrix degradation. Wnt/β-Catenin- related proteins were also significant in the development of KOA as summarized in Figure 6.

## Data Availability

The raw data supporting the conclusions of this article will be made available by the authors, without undue reservation.
